# Inference of genetic relatedness between viral quasispecies from sequencing data

**DOI:** 10.1186/s12864-017-4274-5

**Published:** 2017-12-06

**Authors:** Olga Glebova, Sergey Knyazev, Andrew Melnyk, Alexander Artyomenko, Yury Khudyakov, Alex Zelikovsky, Pavel Skums

**Affiliations:** 1 Computer Science Department, Georgia State University, 25 Park Place NE, Atlanta, 30303 GA USA; 2 Centers for Disease Control and Prevention, 1600 Clifton Rd, Atlanta, 30329 GA USA

**Keywords:** Genetic relatedness, Transmission networks, Outbreaks investigations, Simulation, Clustering

## Abstract

**Background:**

RNA viruses such as HCV and HIV mutate at extremely high rates, and as a result, they exist in infected hosts as populations of genetically related variants. Recent advances in sequencing technologies make possible to identify such populations at great depth. In particular, these technologies provide new opportunities for inference of relatedness between viral samples, identification of transmission clusters and sources of infection, which are crucial tasks for viral outbreaks investigations.

**Results:**

We present (i) an evolutionary simulation algorithm *V*iral *O*utbreak *I*nferen*CE* (*VOICE*) inferring genetic relatedness, (ii) an algorithm MinDistB detecting possible transmission using minimal distances between intra-host viral populations and sizes of their relative borders, and (iii) a non-parametric recursive clustering algorithm *Re*latedness *D*epth (*ReD*) analyzing clusters’ structure to infer possible transmissions and their directions. All proposed algorithms were validated using real sequencing data from HCV outbreaks.

**Conclusions:**

All algorithms are applicable to the analysis of outbreaks of highly heterogeneous RNA viruses. Our experimental validation shows that they can successfully identify genetic relatedness between viral populations, as well as infer transmission clusters and outbreak sources.

## Background

Inferring transmission clusters, transmission directions, and sources of outbreaks from viral sequencing data are crucial for viral outbreaks investigation. Outbreaks of RNA viruses, such as Human Immunodeficiency Virus (HIV) and Hepatitis C virus (HCV), are particularly dangerous and pose a significant problem for public health. It is well known that genomes of RNA viruses mutate at extremely high rates [1]. As a result, RNA viruses exist in infected hosts as populations of closely related variants called *quasispecies* [2, 3]. However, only recently with the progress. Consequently, a contribution of sequencing technologies to molecular surveillance of viral disease epidemic spread becomes more and more substantial [10, 11].

Computational methods can be used to infer transmission characteristics from sequencing data. The first question usually is whether two viral populations belong to the same outbreak. The methods typically utilize the simple observation that all samples from the same outbreak are genetically related, so they use some measure of genetic relatedness as a predictor for epidemiological relatedness [10–12].

The second question is which samples constitute isolated outbreaks. For this purposes, we define a transmission cluster as a connected set of genetically related viral populations. The third questions we address in this article is “Who is the source of infection?”. This questions is the most difficult to answer, and there were only a few attempts to do it computationally using solely genomic data [13] without invoking additional epidemiological information [14]. To the best of our knowledge, there is still no freely available computational tool for this problem.

Computational methods for detection of viral transmissions and inference of transmission clusters are often consensus-based, i.e. they analyze only a single representative sequence per intra-host population (for example, consensus sequence). Such methods assign two hosts into one transmission cluster, if the distances between corresponding sequences do not exceed a predefined threshold [10, 11]. Although consensus-based methods proved to be useful, they do not take into account intra-host viral diversity. Inclusion of whole intra-host populations into analysis is important, because minor viral variants are frequently responsible for transmission of RNA viruses [15, 16].

Recently published computational approach (further referred to as MinDist) [12] uses the minimal genetic distance between sequences of two viral populations as a measure of genetic relatedness of intra-host viral populations. Since minimal genetic distances between different pairs of populations can be achieved on various pairs of sequences, this approach takes into account intra-host diversity.

However, both consensus-based and MinDist approaches have further limitations. First of all, they do not allow to detect directions of transmissions, which is crucial for detection of outbreak sources and transmission histories. Secondly, distance thresholds utilized by both approaches could be derived from analysis of limited or incomplete experimental data and highly data- and situation-specific, with different viruses or even different genomic regions of the same virus requiring specifically established thresholds.

In this paper, we address the above limitations by proposing two novel algorithms *ReD* and *VOICE*, as well as by suggesting an improvement of the MinDist algorithm. The new algorithms allow to infer important epidemiological characteristics, including genetic relatedness, directions of transmissions and transmission clusters.

- *Re*latedness *D*epth (*ReD*) method uses clustering-based analysis of intra-host viral populations. It is a non-parametric algorithm, so it does not rely on any virus-specific threshold values to predict epidemiological characteristics.
- *V*iral *O*utbreak *I*nferen*CE* (*VOICE*) is a simulation-based method which imitates viral evolution as a Markov process in the space of observed viral haplotypes
- MinDistB method is a modification of MinDist [12], which takes into account the sizes of relative borders of each pair of viral populations.

The proposed algorithms were validated on the experimental data obtained from HCV outbreaks. Comparative results suggest that our methods are efficient in epidemiological characteristics inference.

## Methods

### Relatedness depth (*ReD*) algorithm

**Fig. 1:**
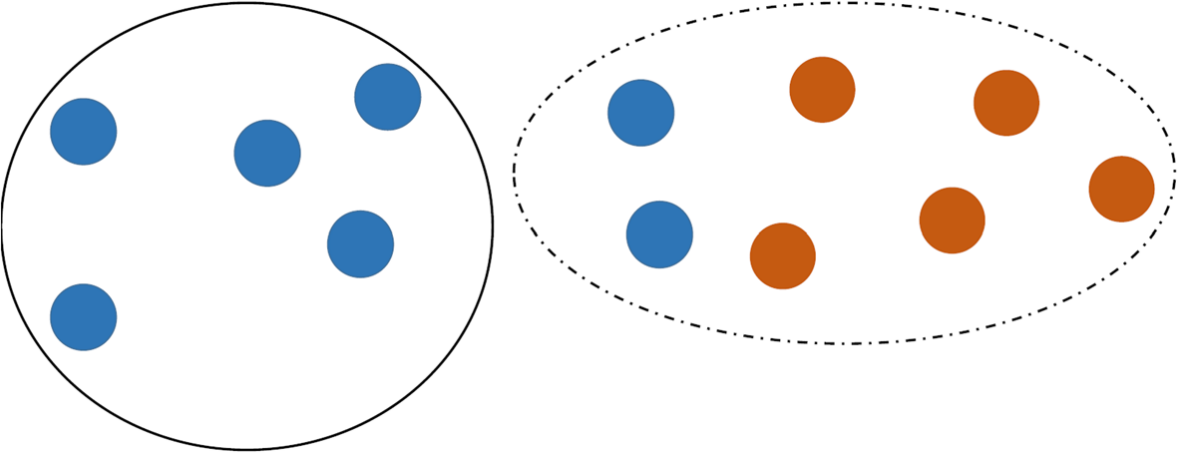
*k*-clustered intersection of two viral populations (blue and red). Union of populations is partitioned into *k*=2 clusters (dashed and solid). Dashed cluster is the *k*-clustered intersection. Direction of transmission is from the blue population to the red population

*ReD* is a deterministic algorithm based on deterministic hierarchical clustering. The key concept of this method is a *k-clustered intersection* of viral populations (we used similar idea previously for combinatorial pooling [17]). For two sets of viral sequences *P*_1_ and *P*_2_, their *k*-clustered intersection $P_{1} \overline {\cap } P_{2}$ is calculated as follows:

1. Partition the union *P*_1_∪*P*_2_ into *k* clusters *C*_1_,...,*C*_*k*_;
2. $P_{1} \overline {\cap } P_{2} = \bigcup \limits _{i\in B} C_{i}$, where *B*={*i*∈{1,...,*k*}:*C*_*i*_∩*P*_1_≠*∅*,*C*_*i*_∩*P*_2_≠*∅*}, i.e. $P_{1} \overline {\cap } P_{2}$ is the union of clusters, which contain sequences from both *P*_1_ and *P*_2_ (see Fig. 1);

The parameter *k* is a *scale* of clustering. In particular, populations *P*_1_ and *P*_2_ are *separable*, if $P_{1} \overline {\cap } P_{2} = \emptyset $, while the fact that $P_{1} \overline {\cap } P_{2} \ne \emptyset $ indicates that they may be genetically related. In the most extreme case $P_{1} \overline {\cap } P_{2} = P_{1}\cup P_{2}$, i.e. populations are *completely inseparable* under the scale *k*.

The degree of confidence that the samples are genetically close is represented by the *relatedness depth* *d*(*P*_1_,*P*_2_), which is calculated by Algorithm 1. Simply speaking, Algorithm 1 tries to recursively separate populations *P*_1_ and *P*_2_. At each iteration, *k*-clustered intersection is calculated. If two populations are separable, then the algorithm stops. Otherwise, it continues the separation of sequences from *P*_1_ and *P*_2_ within their *k*-clustered intersection. The separation depth is a depth of this recursion. It is possible that at some iterations of Algorithm 1 two populations are completely inseparable under a current clustering scale. In this case, the scale *k* is increased and *k*-clustered intersection is recalculated. The initial value of *k* used by Algorithm 1 is *k*=2.



*k*-clustered intersections depend on a clustering method. Our implementation uses a hierarchical clustering based on neighbor-joining tree (as implemented in Matlab (MathWorks, Natick, MA)). The algorithm utilizes a standard Jukes-Cantor distance which is based on the simplest substitution-based evolutionary model.

Clustered intersections also allow for estimating the direction of transmissions. It is reasonable to assume that if two hosts share a population, then a host with more heterogeneous population is more likely to be the transmission source [18]. Formally, if $I = P_{1} \overline {\cap } P_{2}$, *P*_1_⊆*I* and *P*_2_ ∖ *I*≠*∅*, then we assume that probable transmission direction is from *P*_2_ to *P*_1_ (see Fig. 1). The direction is defined according to the first occurrence of such situation during execution of Algorithm 1. Note that in some cases direction may not be identified.

Given the collection of viral populations $\mathcal {P} = \{P_{1},...,P_{n}\}$, *ReD* produces the weighted directed genetic relatedness graph *G*=(*V*,*A*,*d*) with $V=\mathcal {P}$. An arc (*P*_*i*_,*P*_*j*_) is in *A* whenever populations *P*_*i*_ and *P*_*j*_ are genetically related, i.e., have sufficiently high relatedness depth; the direction of an arc corresponds to the estimated direction of transmission and its weight to the relatedness depth. Transmission clusters are calculated as weakly connected components of the digraph *G*. To determine transmission clusters, the simplest depth cutoff *T*=1 can be used. In addition, only components containing at least one arc *a* of weight *d*(*a*)≥2 were considered as reliable. For each reliable component, a source *s* of the corresponding outbreak is identified as a vertex with highest eigenvector centrality.

### Viral outbreak inference (*VOICE*) simulation method

*VOICE* is another approach to predict epidemiological characteristics. Unlike *ReD*, it is not deterministic. Instead, it simulates the process of evolution from one viral population (source) into another (recipient) as a Markov process on a union of both populations. VOICE starts evolution from a subset of source sequences called the *border set* and estimates the number of generations required to acquire a genetic heterogeneity observed in the recipient.

Formally, given two sets of viral sequences *P*_1_ and *P*_2_, *VOICE* simulates viral evolution to estimate times *t*_12_ and *t*_21_ needed to cover all sequences from the recipient population under the assumptions that first and second host were sources of infection. Based on the value min{*t*_12_,*t*_21_}, the algorithm decides whether the populations are related. The direction of possible transmission between the related pair is assumed to follow the direction which requires less time.

**Fig. 2:**
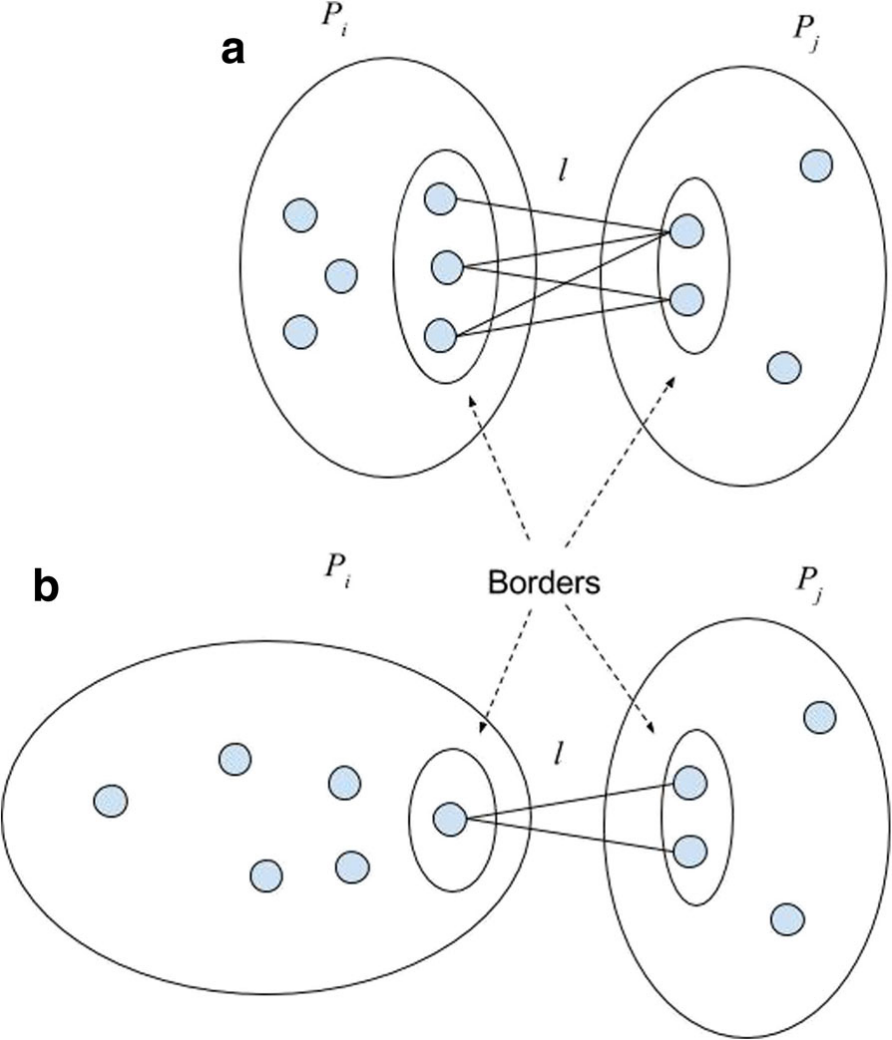
*δ*-Crossing between two viral populations *P*_1_ and *P*_2_ *l*≤*d*(*u*,*v*)+*δ*; (**a**) |*B*_*δ*_|=5; (**b**) |*B*_*δ*_|=2

The simulation starts from the *δ*-*border set* *B*_1_, which contains viral variants that are likely the closest to variants transmitted between *P*_1_ and *P*_2_. It is defined as the set of vertices of *P*_1_ minimizing pairwise Hamming distance *D* between vertices from *P*_1_ and *P*_2_ up to a constant *δ*:

$$B_{1} = \left\{u\in P_{1} : \exists v\in P_{2} ~~ D(u,v) = \min_{x\in P_{1},y\in P_{2}}D(x,y) + \delta \right\} $$

(see Fig. 2). The constant *δ* is a parameter, with the default value 1.

**Fig. 3:**
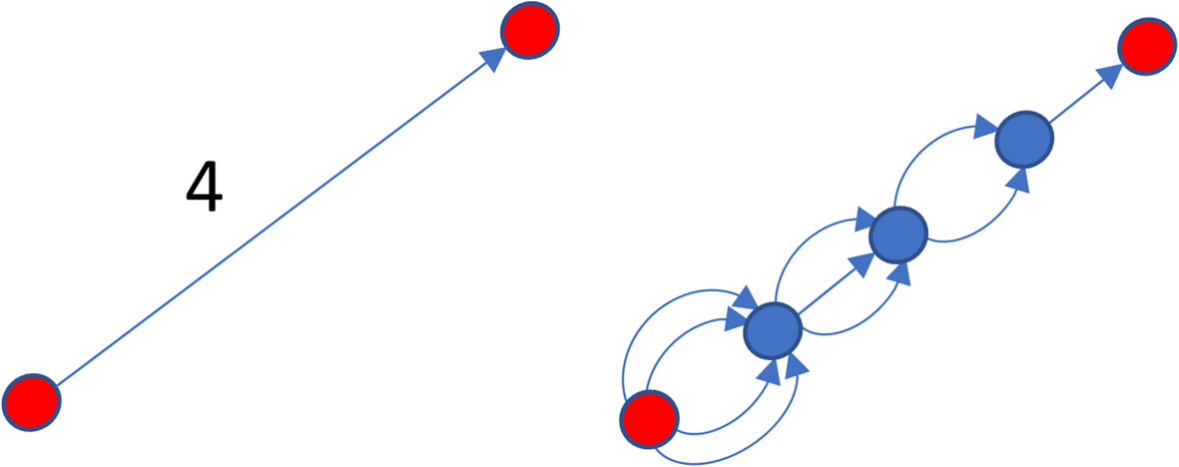
Edge subdividing

The simulated evolutionary process is carried out in the evolutionary space represented by the *variant graph* *G*(*B*_1_,*P*_2_), which is constructed as follows. First, construct a union of all minimal spanning trees of the complete graph on a vertex set *B*_1_∪*P*_2_ with the edge weights equal to Hamming distances between variants (sometimes referred to as a pathfinder network *P* *F* *N* *e* *t*(*n*−1,*∞*) [19, 20]). Then substitute every edge in graph with two directed edges of the same weight. Next, subdivide each edge (*u*_1_,*u*_2_) of weight *w*≥2 with *w*−1 vertices *v*_1_,...,*v*_*w*−1_ and add multiple directed edges as follows: add *w*−1 edges between vertices *u*_1_ and *v*_1_; *w*−2 edges between *v*_1_ and *v*_2_; and so forth as shown on Fig. 3. This model can be explained as follows: to mutate from vertex *u*_1_ to *u*_2_ during simulation, there should occur mutations at *w* positions that are different between *u*_1_ and *u*_2_. During the first step, simulation can mutate any of *w* positions, then any of *w*−1 positions on the second step and so forth.

**Fig. 4:**
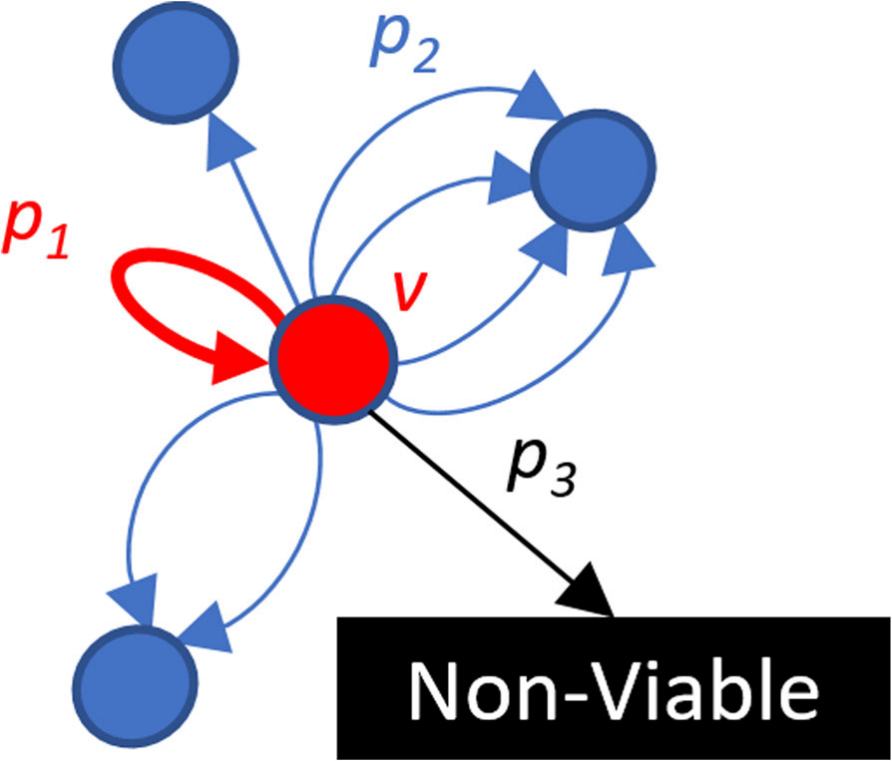
All possible moves of a vertex *v*

The simulation starts from all border vertices *B*_1_ and runs until all the vertices of the population *P*_2_ are reached. At the beginning of the simulation, border vertices get count equal to 1, and the rest of the vertices get count 0. Each tact simulates variants replication by updating vertex counts according to one of the three following scenarios happening with the specified probabilities (see Fig. 4). First, if during replication there are no mutations, then the vertex *v* replicates itself and its count label is incremented. This happens with the probability *p*_1_ (1). Second, the vertex can mutate into one of its neighboring vertices with probability *p*_2_ (see Eq. (2)), in which case the count of the neighbor is incremented. Finally, with probability *p*_3_, vertex does not produce any viable offspring, in which case vertex counts are not changed. If the count of a vertex reaches the maximum allowed variant population size *C*_*max*_, then it is not increased. The probabilities of these scenarios are calculated as follows:

1 $$\begin{array}{@{}rcl@{}} p_{1} & = & (1-3\epsilon)^{L}  \end{array} $$

2 $$\begin{array}{@{}rcl@{}} p_{2} & = & p_{1}\frac{\epsilon}{1-3\epsilon}  \end{array} $$

3 $$\begin{array}{@{}rcl@{}} p_{3} & = & 1 - p_{1} - p_{2}\deg^{-}(v)  \end{array} $$

where *ε* is the mutation rate, *L* is the genome length and deg−(*v*) is an outdegree of a vertex *v*.

Algorithm 2 represents the flow of the method. The time *t*_12_ is computed as the average over *s* simulations. The same procedure is repeated for the opposite direction of the transmission with its border set *B*_2_ and the time *t*_21_ is computed. The value min{*t*_12_,*t*_21_} determines which direction of transmission is more likely.



#### Data normalization

The sizes of observed intra-host viral populations may significantly vary due to sampling and sequencing biases. Since the larger population will require more time to cover, the estimation of *t*_12_ and *t*_21_ could be biased. VOICE avoids such biases by normalizing the intra-host population sizes. The deterministic normalization partitions each viral population into *q* clusters using hierarchical clustering and each cluster is replaced with the consensus of its members. The subsampling normalization randomly chooses *q* sequences from each population. The procedure is repeated *r* times, and the final result is an average over all subsamplings.

#### Identification of genetic relatedness, transmission directions, clusters and sources of outbreaks

Analogously to *ReD*, *VOICE* produces a weighted directed genetic relatedness graph *G*=(*V*,*A*,*w*) with $V=\mathcal {P}$. An arc *P*_*i*_ *P*_*j*_ is in *A* whenever populations *P*_*i*_ and *P*_*j*_ are genetically related, i.e., value min{*t*_*ij*_,*t*_*ji*_} is less than a threshold. Weakly connected components of *G* represent transmission clusters or outbreaks. To determine the source of each outbreak, we build a Shortest Paths Tree (SPT) for every vertex in the corresponding component. The source is estimated as the vertex with an SPT of minimal weight.

### MinDistB method

The method extends the MinDist approach proposed in [12], which defines the distance between viral populations as the minimum Hamming distance between their representatives. The new approach also takes into account sizes of border sets, on which the minimum distance is achieved.

Formally, given an integer *δ* (by default *δ*=1), the *δ*-crossing between populations *P*_1_ and *P*_2_ is the set of pairs of variants (*u*,*v*) from different populations, the Hamming distance *D*(*u*,*v*) between which is within *δ* from the minimum Hamming distance:

$${{\begin{aligned} {}B_{\delta}(P_{1},P_{2})= \left\{(u,v): u \in P_{1}, v \in P_{2}, D(u,v) \le \min_{x\in P_{1},y\in P_{2}}D(x,y) + \delta\right\} \end{aligned}}} $$

(see Fig. 2). Our empirical study shows that in case when the crossing is large (see Fig. 2 a), then the populations are less likely to be related than in case when the borders are small (see Fig. 2 b).

**Fig. 5:**
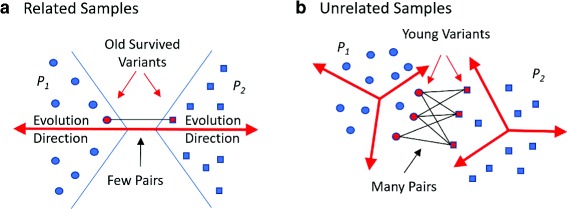
Intuition behind the MinDistB method. **a** Related samples – crossing is between old survived variants. **b** Unrelated samples –crossing is between many young variants which are close to each other by chance

This effect can be intuitively explained. Two related populations likely diverge away from the common ancestor and from each other, and their borders are formed by few old survived variants closest to the common ancestor. Two unrelated populations diverging from two different ancestors may in time reduce minimum distance from each other randomly and closest variants are relatively young and abundant (see Fig. 5).

We define a *δ*-distance between populations *P*_1_ and *P*_2_ as follows:

4 $$ D_{\delta}(P_{1}, P_{2}) = D(P_{1},P_{2}) + c \ln(|B_{\delta} (P_{1},P_{2})|)  $$

where *c*=3 is an empirically chosen constant.

#### Identification of genetic relatedness, transmission clusters and sources of outbreaks

For MinDistB methods, genetic relatedness graph *G*=(*V*,*E*,*w*) is a weighted undirected graph with the vertex set $V=\mathcal {P}$ and an edge of weight *w*_*i*,*j*_ connecting populations *P*_*i*_,*P*_*j*_ whenever *w*_*i*,*j*_=*D*_*δ*_(*P*_1_,*P*_2_) does not exceed a threshold. Transmission clusters are estimated as connected components of the graph *G*. For each transmission cluster its source could be inferred either as a vertex with maximum eigenvector centrality or as a vertex with the shortest paths tree of minimal weight.

## Results and discussions

*ReD*, VOICE and MinDistB were validated using experimental outbreak sequencing data, and their predictions were compared with the previously published MinDist method [12].

### Data sets

We used the benchmark data presented in [12], which is a collection of HCV intra-host populations sampled from 335 infected individuals.

- Outbreak collection contains 142 HCV samples from 33 epidemiologically curated outbreaks reported to Centers for Disease Control and Prevention in 2008–2013. Outbreaks contain from 2 to 19 samples. Epidemiological histories, including sources of infection, are known for 10 outbreaks.
- Collection of 193 epidemiologically unrelated HCV samples.

All viral sequences represent a fragment of E1/E2 genomic region of length 264 bp.

### Prediction of epidemiological characteristics

The proposed methods were used to infer the following epidemiological characteristics:

- genetic relatedness between populations;
- transmission clusters representing outbreaks and isolated samples;
- sources of outbreaks;
- transmission directions between pairs of samples.

Comparison results are collected in Table 1. The variants of VOICE with deterministic and subsampling normalizations are referred to as *V* *O* *I* *C* *E*−*D* and *V* *O* *I* *C* *E*−*S*, and for them we used the normalization constants *q*=10 and *q*=4, respectively. For all VOICE runs, five independent simulations were performed, and the averages over that simulations are reported. For each simulation, VOICE-S performs 50 subsamplings, and the results of the algorithm are averaged over all subsamplings. For MinDist, sources of outbreaks were identified as vertices with highest eigenvector centralities in the corresponding genetic relatedness graphs, since for MinDist this method outperform the shortest path tree-based approach.

**Table 1:** Validation results

Methods	MinDist	MinDistB	*ReD*	VOICE-D	VOICE-S
Relatedness					
Sensitivity, %	90%	92.9%	55.3%	85.2%	86.8%
AUROC	0.992	0.996	N/A	0.993	0.990
Clustering					
Sensitivity, %	100%	100%	96.3%	98.2%	98.2%
Source					
Accuracy, %	50%	40%	90%	80%	90%
Directions					
Accuracy, %	N/A	N/A	87.1%	83.9%	87.1%

#### Genetic relatedness between populations

**Fig. 6:**
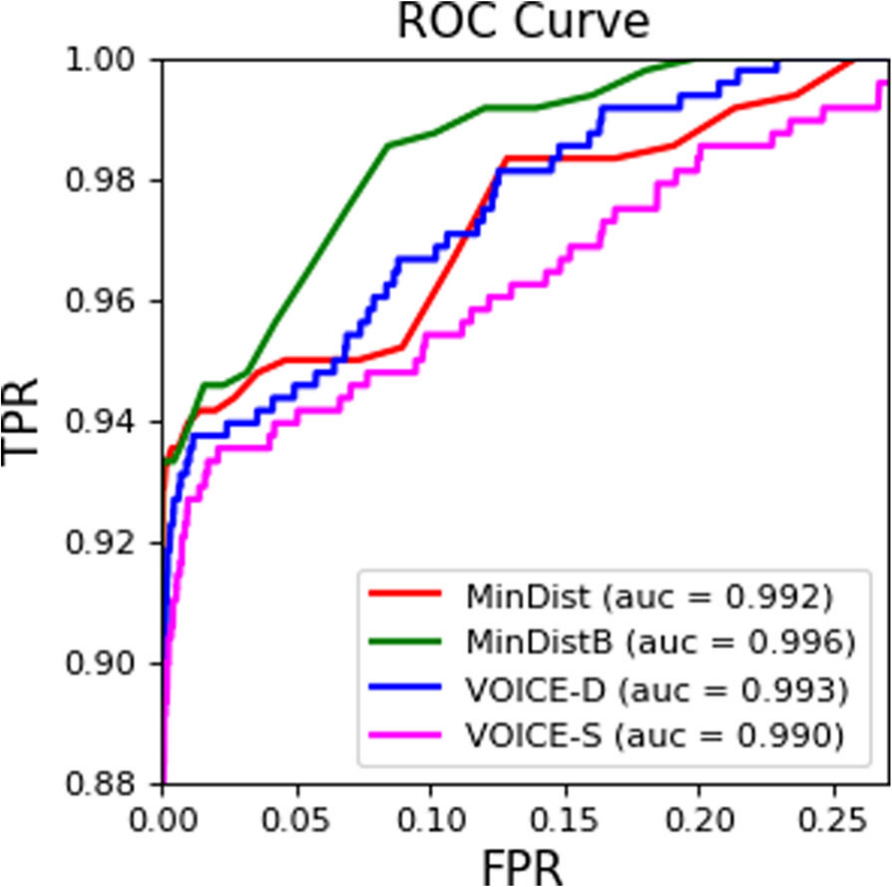
ROC curve for pairs relatedness detection

Viral populations from two samples are genetically related if they belong to the same outbreak and unrelated, otherwise. The genetic relatedness is validated on the union of both collections containing all outbreaks and unrelated samples. There are 55945 pairs of samples, and 479 of them are related. For all algorithms we choose the best thresholds, which produce no false positives, i.e. no unrelated populations are predicted to be related. The values of thresholds *T* are: *R* *e* *D*:*T*=2; *M* *i* *n* *D* *i* *s* *t*:*T*=11; *M* *i* *n* *D* *i* *s* *t* *B*:*T*=28.4; *V* *o* *i* *c* *e*−*D*:*T*=1710; *V* *o* *i* *c* *e*−*S*:*T*=4585. For each method, the sensitivity (i.e. the percentage of detected related pairs) was calculated (Table 1). The highest sensitivity is achieved by MinDistB method. Figure 6 depict ROC curve for the tested methods (*ReD* is not present, since for this method only few viable discrete thresholds are possible). *MinDistB* and *V* *O* *I* *C* *E*−*D* have highest areas under a curve value followed by *MinDist* and *V* *O* *I* *C* *E*−*S*.

#### Detection of transmission clusters

The similarities between true and estimated partitions into transmission clusters were measured using an editing metric [21], which is defined as the minimum number of elementary operations required to transform one partition into another. An elementary operation is either merging (joining of two clusters into a single cluster) or division (partition of a cluster into two clusters) [21]. We calculate sensitivity by normalizing an editing distance *E* by dividing it by the number *N* of elementary operations required to transform trivial partition (i.e. the partition into singleton sets) into the true partition. The number *N* is equal to *n*−*k*, where *n* is the total number of samples and *k* is the number of true clusters:

5 $$ Sensitivity = \frac{E}{n-k} \times 100\%.  $$

Table 1 shows that MinDistB and MinDist demonstrate the highest sensitivity.

#### Source identification

The accuracy of the source identification is defined as the percentage of correctly predicted sources for outbreaks, where the correct sources are known. The Source section of Table 1 shows that the best results are achieved by *ReD* and *V* *O* *I* *C* *E*−*S* which were able to detect sources in 90% of cases. At the same time, MinDist and MinDistB, which are not able to identify transmission directions, were significantly less accurate.

#### Transmission direction

Among tested algorithms, only *ReD* and *VOICE* allows for detection of transmission directions. For that algorithms, percentages of correctly predicted pairs source-recipient were calculated (Table 1). Here the highest accuracy of 87.1*%* was achieved by *ReD* and *V* *O* *I* *C* *E*−*S*.

#### Running time

All tests were performed on PC with DDR3-1333MHz 4 GBx12 RAM and 2 Intel Xeon-X5550 2.67 GHz processors. The fastest algorithms were MinDist and MinDistB, with running times 9 ms for a pair of samples in our dataset. *ReD* requires ∼0.1*s* per pair of samples, While the running time of *VOICE* is ∼35 *s* per pair.

## Conclusions

Currently, a molecular viral analysis is one of the major approaches used for investigations of outbreaks and inference of transmission networks. Although modern sequencing technologies significantly facilitated molecular analysis, providing unprecedented access to intra-host viral populations, they generated novel bioinformatics challenges.

This work proposed three novel algorithms for the investigation of viral transmissions based on analysis of the intra-host viral populations, which allow clustering genetically related samples, infer transmission directions and predict sources of outbreaks. Evaluation of the algorithms on experimental data from HCV outbreaks demonstrated their ability to accurately reconstruct various transmission characteristics. It should be noted, that although *ReD* was proved to be accurate in estimation of transmission clusters, directions and sources, its accuracy of relatedness detection is lower than for other evaluated methods. However, the advantage of this method over other methods is its non-parametricity (i.e. independence from virus-specific and genomic region-specific thresholds), which makes it more universally applicable and extremely useful in situations, when the lack of training data does not allow to establish reliable relatedness thresholds.

The clustering-based *ReD* approach may be further improved using a more scalable clustering similar to the algorithm proposed in [17]. The simulation-based approach *VOICE* presented here may be further improved by incorporating more complex viral evolution models taking into account cell proliferation rate and immune responses against viral variants.

All algorithms are planned to be integrated into the pipeline of cloud-based web-system “Global Hepatitis Outbreak and Surveillance Technology” (GHOST), which is currently being developed by US Centers for Disease Control and Prevention (https://webappx.cdc.gov/GHOST/).
